# The association between psychological capital and sport performance in elite athletes: the chain mediating role of sport self-efficacy and sport self-handicapping

**DOI:** 10.3389/fpsyg.2026.1883522

**Published:** 2026-10-23

**Authors:** Duo Zhang, Weijie Gao, Lumin Liu, Xue Li, Hengyu Li, Huairan Wang, Jin Hwang

**Affiliations:** Physical Education, Jeonbuk National University, Jeonju, Republic of Korea

**Keywords:** elite athletes, psychological capital, sport performance, sport self-efficacy, sport self-handicapping

## Abstract

**Background:**

Elite athletes are continuously exposed to high-intensity training, competitive pressure, and public evaluation. Sport performance is influenced not only by physical fitness and technical-tactical competence but also by psychological resources. As a positive psychological resource, psychological capital may contribute to the maintenance of stable sport performance under stressful conditions. However, existing research has primarily focused on the direct association between psychological capital and sport performance, while the underlying mechanisms, particularly the roles of sport self-efficacy and sport self-handicapping, remain underexamined.

**Objective:**

This study aimed to investigate the association between psychological capital and sport performance among elite athletes and further examine the mediating and chain mediating roles of sport self-efficacy and sport self-handicapping.

**Methods:**

A questionnaire survey was conducted among 410 elite athletes. The instruments included the Psychological Capital Scale, Sport Self-Efficacy Scale, Sport Self-Handicapping Scale, and Sport Performance Scale. Correlation analysis, regression analysis, and Bootstrap chain mediation effect testing were conducted to examine the associations among psychological capital, sport self-efficacy, sport self-handicapping, and sport performance.

**Conclusion:**

The results indicated that psychological capital was not only directly associated with elite athletes' sport performance but also indirectly associated with sport performance through enhanced sport self-efficacy and reduced sport self-handicapping. This study elucidates the psychological mechanism of “enhanced positive capability beliefs—reduced defensive strategies” through which psychological capital is associated with sport performance, providing a theoretical basis for psychological training and performance interventions among elite athletes.

## 1 Introduction

The impact of mental health on competitive performance has become an important topic in international sport psychology (Reardon et al., 2019). This has led to psychological factors gradually emerging as a key entry point for explaining differences in competitive performance. Elite athletes are chronically exposed to multiple stressors, including high-intensity training, competitive pressure, public evaluation, and career development uncertainty. Elite athletes are continuously exposed to multiple stressors, including high-intensity training, competitive pressure, public evaluation, and uncertainty regarding career development. Under such circumstances, strong positive psychological regulation abilities are generally required to maintain stable sport performance in high-pressure environments (Sun and Zhang, 2021). At the same time, in high-level competitive sport, sport performance is determined not only by physical fitness, technical-tactical competence, and training load, but also by psychological resources. Existing research has shown that stable associations exist between psychological variables and sport performance, indicating that psychological resources are important factors in explaining differences in competitive outcomes (Lochbaum et al., 2022).

From the perspective of psychological capital theory, hope, optimism, resilience, and self-efficacy represent interconnected positive psychological resources that help athletes maintain goal orientation, promote recovery from setbacks, strengthen confidence in their capabilities, and maintain engagement and effective adaptation under stressful situations (Luthans et al., 2007; Lai et al., 2020; Yu et al., 2025). This theoretical logic of “upstream resources—proximal psychological and behavioral processes—performance outcomes” is also supported by findings from other performance-related research fields. Cross-domain performance research has similarly demonstrated that upstream resources or individual characteristics often need to be translated into performance outcomes through proximal psychological or behavioral processes and are influenced by situational factors (Shahzad et al., 2023; Cheng et al., 2022; Wang et al., 2024). Therefore, further examining how psychological capital is transformed into sport performance through specific cognitive and behavioral mechanisms among elite athletes has important cross-domain theoretical significance. However, existing research has primarily focused on the associations between psychological capital and burnout, stress, or career development, whereas how psychological capital specifically associates with sport performance itself—particularly the underlying mechanisms among elite athletes—remains underexamined.

From the perspective of the transformation of positive psychological resources into specific competitive behaviors, sport self-efficacy is considered a proximal cognitive mechanism in the present research model. Sport self-efficacy refers to athletes' capability judgments regarding specific training and competition tasks (Anstiss et al., 2018). It is related to, but not identical to, the self-efficacy dimension of psychological capital: psychological capital represents a comprehensive upstream positive psychological resource, whereas sport self-efficacy reflects a situational capability belief oriented toward current competitive tasks (Yang et al., 2023). Previous studies have shown that pre-competition self-efficacy is significantly associated with sport performance and mediates the association between motivational systems and sport performance (Lochbaum et al., 2023; Rogowska et al., 2022). Shao et al. (2023) also provides cross-domain evidence supporting self-efficacy as a proximal mechanism through which upstream influences are translated into specific behaviors. This is consistent with the theoretical arrangement of the present study, which places sport self-efficacy after psychological capital. Specifically, comprehensive positive psychological resources need to be further transformed into situational capability beliefs of “I can complete the current competitive task” before they are more likely to influence subsequent competitive behaviors. However, positive capability beliefs alone are insufficient to fully explain how psychological capital is associated with sport performance. Sport self-handicapping is conceptualized in the present model as a self-protection strategy in response to potential failure threats. Specifically, before performance outcomes occur, athletes may reduce threats to self-worth associated with possible failure by lowering effort, avoiding challenges, creating excuses in advance, or emphasizing external obstacles (Berglas and Jones, 1978; Martin and Brawley, 2002). Therefore, unlike the “positive capability beliefs” represented by sport self-efficacy, sport self-handicapping serves as a mediator representing defensive behavioral tendencies in the present research model. For elite athletes, given the highly public nature of competition outcomes, significant evaluative consequences, and high costs of failure, self-handicapping is more likely to be activated as a short-term self-protective strategy. However, in the long term, this strategy undermines effort and execution, thereby hindering sport performance.

It is worth noting that elite athletes' judgments regarding failure and ability do not occur in completely isolated individual contexts but are embedded within coach feedback, teammate interactions, and team evaluation structures. Related research in team and organizational contexts has shown that social interaction environments can significantly influence individual information processing and performance processes. In elite competitive environments characterized by publicly observable outcomes and high evaluative pressure, sport self-handicapping may reduce threats associated with failure in the short term; however, it may also weaken genuine effort investment and task execution. Social environmental factors, such as leadership feedback, team interactions, and collaborative norms, may further influence individual information processing and performance processes (Guo et al., 2024; Jiang et al., 2012, 2022). Although these studies were not directly conducted in competitive sport contexts, they collectively suggest that performance behaviors depend not only on individual psychological resources but may also be shaped by feedback relationships, interaction structures, and team norms. For elite athletes, this implies that their sport self-efficacy and tendencies toward sport self-handicapping when facing failure threats may also be influenced by coach feedback styles, team relationships, and social evaluation environments. However, although previous studies have indicated a positive association between psychological capital and sport performance, existing evidence has mainly focused on the direct association or correlation between these two variables. The psychological processes through which this positive psychological resource is transformed into specific sport performance remain insufficiently understood. Therefore, the present study integrates psychological capital, sport self-efficacy, and sport self-handicapping into a unified theoretical framework. Specifically, psychological capital first functions as an upstream positive psychological resource that enhances athletes' sport self-efficacy; higher sport self-efficacy further reduces athletes' tendencies toward sport self-handicapping; and reduced sport self-handicapping helps maintain genuine effort, task engagement, and effective behavioral execution, which are ultimately associated with better sport performance. Based on this framework, this study explains the association between psychological capital and sport performance through the sequential process of “enhanced positive psychological resources—enhanced sport capability beliefs—reduced defensive strategies—improved sport performance.”

In addition, the selection of Chinese elite athletes has clear contextual significance. Factors within China's professional competitive sport system, including collective goals, the authority of coaches and managers, interpersonal norms, and social evaluation, may influence athletes' psychological experiences and coping strategies (Si et al., 2015). Research in organizational management, stakeholder relationships, and intercultural competence has also suggested that performance outcomes should be understood within specific institutional and cultural contexts (Tang et al., 2018; Wang et al., 2026; Peng et al., 2026). Studies conducted in Chinese sport contexts have further indicated that cultural orientation, coach support, and sport self-efficacy are associated with performance-related psychological evaluations (Li et al., 2024). Therefore, examining this chain mediation mechanism among Chinese elite athletes may help clarify its cultural relevance and applicability boundaries.

Based on the above theoretical analysis, this study proposes the following hypotheses: H1, psychological capital positively predicts sport performance; H2, sport self-efficacy mediates the association between psychological capital and sport performance; H3, sport self-handicapping mediates the association between psychological capital and sport performance; H4, sport self-efficacy and sport self-handicapping form a chain mediation pathway, such that psychological capital is sequentially associated with higher sport self-efficacy, lower sport self-handicapping, and better sport performance.

## 2 Research hypotheses

### 2.1 The association between psychological capital and sport performance in Elite athletes

Sport performance is influenced by both external physiological factors and internal psychological factors. As a positive psychological resource, psychological capital can affect individuals' cognitive appraisal, emotional regulation, and behavioral engagement in stressful situations. In competitive sport, this positive psychological resource may further associate with athletes' training adaptation and competition performance (Yu et al., 2025). From the perspective of relevant research findings, a significant association exists between marathon runners' positive psychological states and their sport performance, indicating that positive psychological resources may play an important role in performance in endurance events (Larumbe-Zabala et al., 2020). Furthermore, psychological capital is not fixed but can be cultivated and enhanced through training, support, and intervention, which provides a practical foundation for psychological training in competitive sport (Moshir Estekhareh and Doroudian, 2024).

Among the multiple dimensions of psychological capital, optimism is characterized by positive expectations regarding future outcomes and by a tendency for difficulties to be interpreted as temporary and surmountable challenges. Hope is reflected in athletes' sustained motivational orientation toward goals and in the belief that pathways toward goal attainment can be identified; such a positive goal orientation may contribute to the maintenance of training engagement and persistence in competition (Sehrawat et al., 2024). Psychological capital is generally associated with the achievements of high-performing sports teams, as well as in a cohort of students at various universities (Lee et al., 2022); components that have been identified through this research include motivation, perseverance, emotional regulation, resilience, skills, body image, etc. A high-level resilient system will be able to recover from failure more promptly after being damaged (Chrétien et al., 2024). Psychological resources can be regarded as positive resources for a person's mental health, and thus, to reduce fatigue in performance-pressing activities, keep going even after burnout and stay in shape (Glandorf et al., 2023).

In short, the above studies have generally shown a stable positive correlation between psychological capital and sports performance. Higher levels of psychological capital in elite athletes can help them maintain positive expectations, improve recovery capacity, and remain engaged in the face of high-pressure training and competition; thus, they will achieve a better competitive state. Based on the above analysis, it is proposed that Hypothesis H1 holds true: psychological capital positively affects sports performance.

### 2.2 The role of sport self-efficacy in the association between psychological capital and sport performance in Elite athletes

Sport self-efficacy refers to athletes' beliefs in their ability to successfully organize and execute specific training or competition tasks and represents a concrete manifestation of the general self-efficacy concept in competitive sport contexts (Bandura and Wessels, 1997; Anstiss et al., 2018). Different from self-efficacy as one of the core dimensions of psychological capital, sport self-efficacy examined in this study emphasizes the task-specific capability judgments formed by athletes based on specific training and competition demands. Higher levels of psychological capital are generally associated with a greater tendency to maintain positive expectations, strengthen perceptions of control, and sustain engagement under difficult circumstances. In contrast, lower levels of psychological capital are more likely to be accompanied by negative emotional experiences and avoidance tendencies. The dimensions of psychological capital do not operate independently; rather, synergistic effects are formed during processes of goal pursuit and stress coping (Waddington, 2023). Psychological capital contributes to the integration and coordination of positive psychological resources and may strengthen the connections among positive resources such as self-efficacy, hope, and resilience through the enhancement of goal-directed confidence and positive coping tendencies (Bai and Ma, 2025). In competitive tennis contexts, stronger positive psychological resources have been found to facilitate faster recovery of attentional focus following performance errors and the maintenance of emotional stability, thereby strengthening confidence in subsequent sport performance (Harris et al., 2021).

Sport self-efficacy is considered an important variable influencing athletes' behavioral choices, effort levels, persistence, and emotion regulation, and is closely related to competitive performance (Wang et al., 2025). Self-efficacy can mediate the association between motivational orientation and sport performance, with higher levels of self-efficacy generally associated with better sport performance (Rogowska et al., 2022).

In summary, psychological capital may further promote focused engagement, strategic adjustment, and stable performance by enhancing athletes' capability beliefs in training and competition, thereby indirectly associating with better sport performance. Based on this, Hypothesis H2 is proposed: Psychological capital is positively associated with sport performance through the enhancement of sport self-efficacy.

### 2.3 The role of sport self-handicapping in the association between psychological capital and sport performance in Elite athletes

Self-handicapping refers to a defensive strategy in which individuals actively create, claim, or accept obstacles in performance situations to reduce self-threat in the event of failure and attribute negative outcomes to external factors (Leary, 2019). Regarding the formation mechanisms of self-handicapping, existing research primarily explains it through two pathways: first, as a strategy to protect self-worth (Berglas and Jones, 1978), and second, as a means of regulating external evaluations and social impressions (Kolditz and Arkin, 1982). The positive psychological resources represented by psychological capital help athletes maintain a sense of control, goal persistence, and recovery from setbacks, whereas self-handicapping manifests more as a defensive response aimed at protecting self-esteem. When positive psychological resources such as resilience are weak, athletes are more likely to resort to self-handicapping behaviors when facing difficulties or failures (Cai et al., 2025). Overall, psychological capital and sport self-handicapping are generally negatively associated. Higher levels of psychological capital may reduce the likelihood of athletes adopting self-handicapping strategies in high-pressure situations by enhancing hope, optimism, resilience, and self-efficacy; conversely, frequent self-handicapping may also weaken individuals' positive psychological resources, creating a detrimental cycle. In competitive sport, given the uncertainty of competition outcomes, high competitive pressure, and the fact that performance is often under public scrutiny, elite athletes are particularly prone to self-handicapping tendencies in high-pressure situations. When athletes anticipate failure or doubt their performance outcomes, they are more likely to reduce psychological threat from failure by lowering effort, making excuses in advance, or externalizing attributions (Török et al., 2022).

In summary, psychological capital may have an inhibiting effect on sport self-handicapping, whereas higher levels of sport self-handicapping are generally detrimental to athletes' training engagement and competitive performance. The findings suggest that psychological capital may reduce athletes' likelihood of adopting sport self-handicapping strategies under failure threats by enhancing positive expectations, perceived control, recovery from setbacks, and task-specific confidence. Meanwhile, sport self-handicapping may be further associated with poorer sport performance by reducing genuine effort, increasing avoidance tendencies, and interfering with task execution. Accordingly, Hypothesis H3 is proposed: psychological capital is positively associated with sport performance through the reduction of sport self-handicapping.

### 2.4 The association between sport self-efficacy and sport self-handicapping

Sport self-efficacy and sport self-handicapping are two important variables in the psychological regulation process of athletes, and a significant negative association may exist between them. Self-efficacy is considered an important psychological factor influencing individuals' behavioral choices and persistence: the higher the level of self-efficacy, the more likely individuals are to adopt positive behaviors and maintain sustained engagement, whereas lower capability beliefs are more likely to be accompanied by avoidance and defensive reactions. In sport contexts, this suggests that higher sport self-efficacy may inhibit the occurrence of self-handicapping behaviors to a certain extent (Tang and Guo, 2021). In a study on return to sport following anterior cruciate ligament reconstruction, Burland et al. (2018) found that higher task confidence helped reduce avoidance tendencies arising from doubt, worry, and low sense of control, providing indirect support for understanding how sport self-efficacy inhibits self-handicapping. In terms of mechanisms, higher sport self-efficacy may reduce athletes' tendency to adopt self-handicapping strategies by altering their interpretation of failure, enhancing their sense of action control, and maintaining achievement motivation. At the same time, prolonged self-handicapping will not be conducive to the self's improvement and will further diminish a sense of self-control. Therefore, sport self-efficacy and sport self-handicapping have opposite functions: the former is a sense of capability and active coping, while the latter is defensive self-protection. The two indicators may work together in the process of psychological regulation for athletes to affect their sports performance jointly. In short, close to a psychological capital is present among elite athletes in terms of self-efficacy in sport. Sport self-efficacy is also related to sport self-handicapping, and sport self-handicapping in turn affects sports performance. Therefore, hypothesis H4 is proposed: psychological capital positively affects sport performance through the chain mediation path of “increased sport self-efficacy and reduced sport self-handicapping. The hypothesized model is shown in Figure 1.

**Figure 1:**
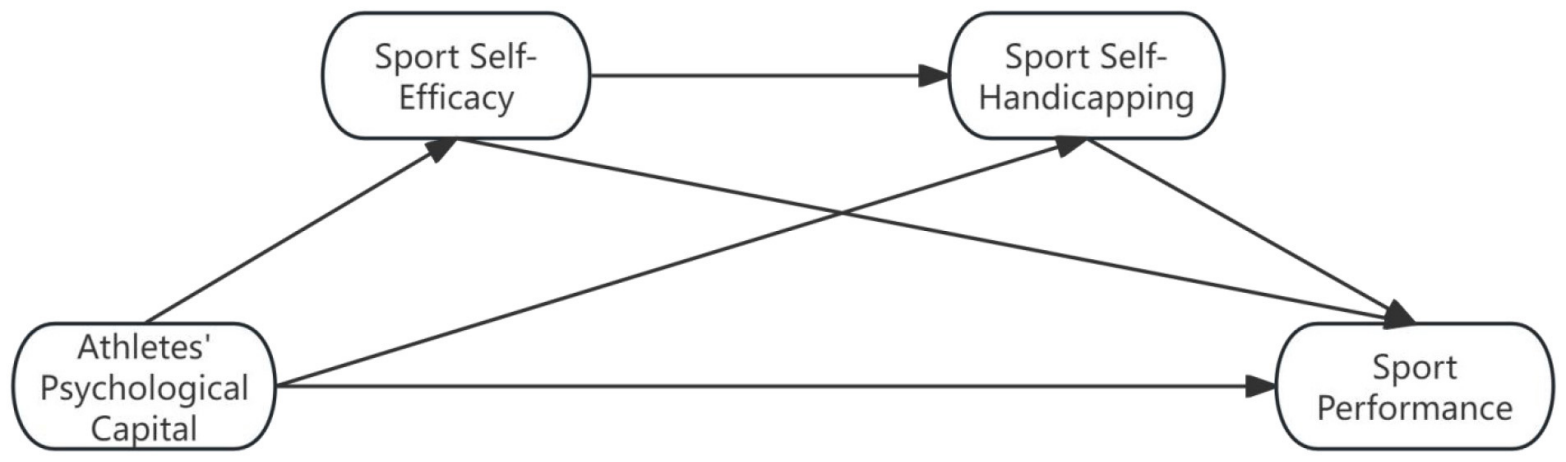
The conceptual model proposed in this study.

## 3 Research methods

### 3.1 Research participants

This study adopted a non-probability sampling approach to recruit active elite athletes from provincial-level and above professional sports teams in Henan, Shandong, Jilin, Shaanxi, and Hebei provinces. After obtaining permission from the relevant professional sports teams, researchers introduced the study information to eligible athletes with the assistance of coaches or team managers. Participation was entirely voluntary and determined by the athletes themselves. Because elite athletes represent a specific population with clear professional training and competitive qualifications, and their daily training and management are characterized by strong organizational structures, this study did not conduct probability random sampling or individual random selection from a nationwide list of elite athletes. Therefore, the sampling approach was primarily used to obtain participants who met the study criteria and possessed authentic professional competitive experience. A total of 440 questionnaires were distributed, 425 were returned, resulting in a response rate of 96.5%. Among them, 410 questionnaires were considered valid, yielding a valid response rate of 96.47%. All participants were active professional athletes who had continuously participated in official provincial-level or higher competitions during the previous 2 years. Participants were required to meet the following inclusion criteria: (1) currently registered as active athletes; (2) having participated in official provincial-level or higher competitions within the previous 2 years; (3) having no severe sports injuries or known serious mental disorders; and (4) voluntarily signing informed consent forms. The study protocol was approved by the Ethics Committee of Henan Normal University (Approval No.: HNSD-2025-15-11). To reduce potential sample bias caused by a single region or team source, this study included athletes from different provincial-level and above professional sports teams across five provinces and applied consistent inclusion criteria and standardized data collection procedures. As nationwide probability sampling was not conducted, this sample should not be considered representative of all Chinese elite athletes. Therefore, the findings of this study are mainly applicable to athletes with similar professional training backgrounds and formal competition experience.

### 3.2 Research instruments

To ensure the applicability of the measurement instruments among Chinese elite athletes, this study prioritized the use of established scales that were either available in Chinese versions or developed/adapted by Chinese scholars. The Psychological Capital Scale, Sport Self-Efficacy Scale, and Sport Self-Handicapping Scale were all adopted from existing Chinese versions, and the Sport Performance Scale was also based on an available Chinese version. Therefore, additional cross-language translation procedures from the original English versions were not conducted in this study. Considering that the original items of the Psychological Capital Scale were not specifically developed for competitive sport contexts, this study contextualized several item expressions to training and competition settings while maintaining the original theoretical dimensions, core meanings, and scoring procedures, thereby improving its applicability to elite athletes. To further evaluate the applicability of each measurement instrument among Chinese elite athletes, this study assessed the instruments from three aspects: first, the language version of each scale and its compatibility with Chinese culture and competitive sport contexts; second, evidence from the original scale development studies and previous applications or validations in similar populations; and third, the internal consistency reliability and structural validity re-examined within the present study sample. It should be noted that the previous validation samples of different scales were not completely identical to the 410 Chinese elite athletes included in this study. Therefore, previous studies mainly provided external psychometric evidence, whereas Cronbach's α coefficients and confirmatory factor analysis results obtained from the present sample served as direct evidence for evaluating the applicability of these instruments in the current study population.

#### 3.2.1 Athletes' psychological capital scale

The Chinese version of the Positive Psychological Capital Questionnaire (PPCQ) developed by Zhang et al. (2010) was used to measure elite athletes' psychological capital. The questionnaire consists of four dimensions: self-efficacy, resilience, hope, and optimism, with a total of 26 items. Responses were rated on a 7-point Likert scale, with higher scores indicating higher levels of psychological capital. Zhang et al. conducted exploratory and confirmatory factor analyses of the questionnaire among Chinese university students. The results supported the four-dimensional structure and higher-order structure of psychological capital, and demonstrated good structural validity and internal consistency reliability, indicating that the instrument has a sound psychometric foundation within the Chinese cultural context. However, the original validation sample primarily consisted of university students rather than competitive athletes; therefore, previous evidence cannot be directly considered equivalent to its applicability among elite athletes. Considering the contextual specificity of elite athletes in terms of training pressure, competitive evaluation, and task objectives, this study moderately contextualized several general item expressions based on daily training and competition situations, while maintaining the original core meanings, dimension attribution, and scoring procedures of the items. This approach aimed to preserve the theoretical structure of the original questionnaire while improving the comprehensibility and content relevance of the items within competitive sport contexts. To further examine the applicability of the questionnaire among Chinese elite athletes, this study additionally assessed its internal consistency reliability and structural validity. The Cronbach's α coefficient of the scale in this study was 0.863; thus, it had good internal consistency reliability among the subjects. The structural validity fit indices were as follows: χ^2^/*df* = 0.991, GFI = 0.950, AGFI = 0.940, RMSEA = 0.024, CFI = 0.981, NFI = 0.932, and IFI = 1.000, and good structural validity was shown by the scale in the current study.

#### 3.2.2 Sport self-efficacy scale

This study used the Chinese version of the Sport Self-Efficacy Scale developed by Wei et al. (2008). Based on Bandura's self-efficacy theory and developed specifically for athletes' training and competition contexts, the scale consists of two dimensions: training efficacy and competition efficacy. It includes 15 items rated on a 5-point Likert scale. Because the items directly address training tasks, competition difficulties, tactical execution, physical preparation, and competitive pressure, the measured constructs and contexts are highly consistent with the characteristics of the elite athletes included in this study. In the original scale development study, the internal consistency reliability of the scale was examined. The total scale and its two dimensions, competition efficacy and training efficacy, demonstrated acceptable reliability levels, indicating that the scale can provide a relatively stable assessment of capability beliefs in sport contexts. Meanwhile, this scale is a Chinese measurement instrument directly developed by Chinese scholars for sport contexts. Therefore, it shows good alignment with the present sample in terms of language and competitive sport contexts, and additional cross-language translation was not required. The Cronbach's α coefficient of the scale in this study was 0.992, which shows good internal consistency reliability of the study sample. The structural validity indices were as follows: χ^2^/*df* = 1.096, GFI = 0.970, AGFI = 0.959, RMSEA = 0.015, CFI = 0.995, NFI = 0.945, and IFI = 0.995; thus, the scale had good structural validity.

#### 3.2.3 Sport self-handicapping

This study used the Sport Self-Handicapping Scale revised by Sun and Zhang (2008) to measure the level of sport self-handicapping among elite athletes. The scale consists of 10 items rated on a 5-point Likert scale, with higher scores indicating stronger tendencies toward sport self-handicapping in training or competition contexts. Compared with self-handicapping measures developed in general domains, this scale directly considers failure threats, performance evaluation, and self-protection behaviors within competitive sport contexts as its measurement background. Therefore, it demonstrates greater content relevance to the competitive situations examined in this study. The original study by Sun and Zhang focused on individual athletes, and subsequent research among high-level competitive athletes has further indicated that self-handicapping represents an important psychological variable in studies of competitive stress and athletes' capability beliefs. Therefore, this instrument and the construct it measures have an empirical basis for application among Chinese athletes. However, previous studies have mainly focused on individual-sport athletes or specific athlete populations. The Cronbach's α coefficient for this scale was 0.871, indicating good internal consistency within the study sample. The structural validity fit indices are as follows: χ^2^/*df* = 1.409, GFI = 0.977, AGFI = 0.964, RMSEA = 0.032, CFI = 0.987, NFI = 0.956, and IFI = 0.987, indicating that the scale has good structural validity.

#### 3.2.4 Sport performance scale

This study used the Sport Performance Scale originally developed by Rees et al. (2000) and adapted into Chinese by Tang (2020). The scale includes seven dimensions—plan execution, loss of composure, determination, worry, fatigue, flow, and effective tactics—comprising 28 items. A 5-point Likert scale was used, with 1 indicating “strongly disagree” and 5 indicating “strongly agree.” In this study, sport performance was measured using a self-reported Sport Performance Scale. This measure primarily reflects athletes' subjective perceptions of their performance based on their own training and competition experiences, including plan execution, emotional control, determination, fatigue, fluency, and tactical execution. Therefore, it represents perceived or self-reported sport performance rather than objective competition outcomes, such as competition rankings, competition results, win-loss outcomes, or objective technical statistics.

This scale has been directly validated among competitive athletes. Rees et al. (2000) conducted confirmatory factor analysis of the measurement model using a sample of 155 full-time tennis players. The results supported the seven dimensions of sport performance and indicated that related performance dimensions could differentiate winners and losers, providing evidence for the structural validity and criterion-related validity of the instrument among competitive athletes. Subsequently, the scale was translated and adapted for use in Chinese contexts, providing linguistic and cultural support for its application among Chinese athletes. However, previous validation and Chinese applications of this instrument have primarily focused on specific sports, such as tennis, whereas the present study included diverse elite athletes from both individual and team sports. Therefore, this study did not assume that the original factor structure could be unconditionally generalized; instead, the internal consistency reliability and construct validity of the scale were re-examined within the current sample. The overall Cronbach's α coefficient for this scale was 0.949, indicating good internal consistency within the study sample. The structural validity fit indices are as follows: χ^2^/*df* = 1.198, GFI = 0.937, AGFI = 0.923, RMSEA = 0.022, CFI = 0.986, NFI = 0.922, and IFI = 0.986, indicating that the scale has good structural validity.

### 3.3 Data collection

Data were collected using an on-site questionnaire survey. The complete questionnaire consisted of demographic information and four measurement scales, with a total of 79 scale items. After confirming the inclusion criteria and obtaining informed consent, researchers used standardized instructions to explain the purpose of the study, completion requirements, and principles of anonymity and confidentiality. Participants completed the questionnaire independently based on their actual training and competition experiences. The questionnaires were supervised and collected on site by trained psychology graduate students. They provided explanations only regarding completion procedures and did not induce specific responses. Coaches and team managers were not involved in questionnaire completion. No monetary, gift, or other material incentives were provided for participation in this study.

### 3.4 Statistical analysis

First, descriptive statistics were conducted to examine participants' demographic characteristics and the distributional characteristics of the main study variables. Pearson correlation analysis was used to examine the bivariate associations among psychological capital, sport self-efficacy, sport self-handicapping, and sport performance. Based on these analyses, multiple regression analyses were further conducted to examine the path relationships among variables, providing path coefficients and model explained variance for subsequent indirect effect analyses. To control for potential confounding effects of demographic factors, sex and age were included as covariates in both component path regression analyses and the chain mediation model. Sex was dummy coded (female = 0, male = 1), and age was entered into the model as a continuous variable. In addition, sport type (individual/team sports) was recorded. Because this binary classification does not adequately capture heterogeneity across specific sports, and the purpose of this study was not to compare sport types, it was not included in the primary models. Its potential influence is discussed in the Discussion and Limitations sections.

To further evaluate the practical effect sizes of regression predictors, this study calculated local Cohen's *f*^2^ effect sizes for each regression coefficient in addition to reporting standardized regression coefficients (β), model explained variance (*R*^2^), and statistical significance. Local *f*^2^ reflects the independent explanatory contribution of a target predictor to the dependent variable after controlling for other predictors and covariates within the same model. According to Cohen's (2013) traditional criteria, *f*^2^ values of 0.02, 0.15, and 0.35 can be considered reference thresholds for small, medium, and large effects, respectively. These criteria were used only to assist in the interpretation of effect sizes.

Considering that the chain mediation model included multiple predictors and that sport self-efficacy and sport self-handicapping were correlated to some extent, multicollinearity among predictors was examined before conducting regression and mediation analyses. Variance inflation factor (VIF) and tolerance values were used for diagnosis, with tolerance representing the reciprocal of VIF. According to commonly used diagnostic principles in multiple regression analysis, substantially elevated VIF values and low tolerance values indicate potential multicollinearity risks. In this study, VIF < 5 and tolerance > 0.20 were used as conservative reference criteria, and specific values were considered to determine whether serious collinearity problems existed among predictors (O'brien, 2007).

Chain mediation analysis was conducted using PROCESS Model 6 (Hayes, 2022), with psychological capital as X, sport self-efficacy as M1, sport self-handicapping as M2, and sport performance as Y. Sex and age were included as covariates. The model estimated a1 (X → M1), a2 (X → M2), d21 (M1 → M2), b1 (M1 → Y), b2 (M2 → Y), and the direct effect c′, and tested three specific indirect effects: a1b1 (H2), a2b2 (H3), and a1d21b2 (H4). Considering that the sampling distribution of indirect effects may deviate from normality, 95% confidence intervals were generated using 5,000 bootstrap samples based on MacKinnon et al. (2004) and Preacher and Hayes (2008). Indirect effects were considered significant when the confidence interval did not include zero. Given the cross-sectional design of this study, the serial mediation findings only indicate that the data are consistent with the hypothesized indirect association structure and cannot establish temporal order or causal relationships.

## 4 Results analysis

### 4.1 Demographic information

The final sample included 410 elite athletes from provincial-level and above professional sports teams in China. Complete demographic information was available for all participants. The sample consisted of 224 males (54.6%) and 186 females (45.4%). Participants ranged in age from 18.0 to 32.0 years, with a mean age of 22.80 years (SD = 3.70), a median age of 22.50 years, and an interquartile range of 19.53–25.60 years. According to sport type, 214 athletes (52.2%) participated in individual sports and 196 athletes (47.8%) participated in team sports. The mean age was 22.51 years (SD = 3.62) for individual-sport athletes and 23.11 years (SD = 3.77) for team-sport athletes. Among individual-sport athletes, 108 were males (50.5%) and 106 were females (49.5%); among team-sport athletes, 116 were males (59.2%) and 80 were females (40.8%). Participants had 6.0–15.0 years of training experience, with a mean training duration of 9.20 years (SD = 2.60), a median of 9.00 years, and an interquartile range of 6.90–11.10 years. The mean training duration was 9.22 years (SD = 2.72) for individual-sport athletes and 9.18 years (SD = 2.47) for team-sport athletes. Regarding athlete classification, 46 participants (11.2%) were international-level athletes, 130 participants (31.7%) were national-level athletes, and 234 participants (57.1%) were first-class athletes. The detailed demographic characteristics are presented in Table 1. Sport type was reported descriptively to present the composition of the sample. In the subsequent primary statistical models, age and sex were included as covariates, whereas sport type was not included as a covariate.

**Table 1:** Demographic information on participants.

Characteristics	Total (*N* = 410)	Individual sports (*n* = 214)	Team sports (*n* = 196)
Sex, *n* (%)			
Male	224 (54.6)	108 (50.5)	116 (59.2)
Female	186 (45.4)	106 (49.5)	80 (40.8)
Age (years)			
Mean ± SD	22.80 ± 3.70	22.51 ± 3.62	23.11 ± 3.77
Median (IQR)	22.50 (19.53–25.60)	22.05 (19.43–25.15)	23.10 (19.68–25.90)
Range	18.0–32.0	18.0–32.0	18.0–32.0
Training years (years)			
Mean ± SD	9.20 ± 2.60	9.22 ± 2.72	9.18 ± 2.47
Median (IQR)	9.00 (6.90–11.10)	9.00 (6.53–11.28)	9.10 (7.00–11.00)
Range	6.0–15.0	6.0–15.0	6.0–15.0
Athlete classification, *n* (%)			
International-level athletes	46 (11.2)	28 (13.1)	18 (9.2)
National-level athletes	130 (31.7)	67 (31.3)	63 (32.1)
First-class athletes	234 (57.1)	119 (55.6)	115 (58.7)

Categorical variables are presented as *n* (%), and percentages in the individual sports and team sports columns represent within-group percentages. Continuous variables are presented as mean ± standard deviation (M ± SD), median and interquartile range (IQR), and minimum–maximum range. *N* = 410.

### 4.2 Common method bias test

This study used Harman's single-factor test to examine common method bias. All items measuring the variables were entered together into a factor analysis. The results showed that there were 9 factors with eigenvalues greater than 1, and the variance explained by the first common factor was 32.236%, which is below the critical criterion of 40% (Tang and Wen, 2020). However, it should be noted that Harman's single-factor test itself can only provide a preliminary and rough assessment and cannot completely rule out the potential influence of common method bias. Particularly in cross-sectional questionnaire studies, participants' social desirability responses, the same measurement context, and similar scale formats may still introduce some interference into the associations among variables. Therefore, the results of this study should not be fully interpreted as being free from common method bias, but rather as indicating that no dominant common method bias was observed under the current sample and measurement conditions.

### 4.3 Correlation analysis of variables

As shown in Table 2, elite sport Psychological Capital was significantly positively associated with sport self-efficacy and sport performance. Specifically, psychological capital was significantly positively associated with sport self-efficacy (*r* = 0.495, *p* < 0.01), and psychological capital was significantly positively associated with sport performance (*r* = 0.595, *p* < 0.01). Psychological capital was significantly negatively associated with sport self-handicapping (*r* = −0.529, *p* < 0.01). Sport self-efficacy was significantly positively associated with sport performance (*r* = 0.602, *p* < 0.01) and significantly negatively associated with sport self-handicapping (*r* = −0.496, *p* < 0.01). Sport self-handicapping was significantly negatively associated with sport performance (*r* = −0.637, *p* < 0.01). These results provide preliminary support for subsequent testing of the mediating roles of sport self-efficacy and sport self-handicapping in the association between psychological capital and sport performance.

**Table 2:** Means, standard deviations, and correlation coefficients among variables.

Variables	M	SD	Sport psychological capital	Sport self-efficacy	Sport self-handicapping	Sport performance
Sport psychological capital	5.344	0.661	1			
Sport self-efficacy	3.845	0.484	0.495^**^	1		
Sport self-handicapping	2.167	0.513	−0.529^**^	−0.496^**^	1	
Sport performance	3.951	0.451	0.595^**^	0.602^**^	−0.637^**^	1

*N* = 410; ^*^p < 0.05, ^**^p < 0.01.

### 4.4 The association between elite sport psychological capital and sport performance

According to the mediation analysis procedure described in Section 3.4, this study first examined the direction and strength of each component path in the chain mediation model through regression analyses before formally testing the Bootstrap indirect effects. Model 1 examined the total effect of psychological capital on sport performance; Model 2 examined the association between psychological capital and sport self-efficacy; Model 3 simultaneously included psychological capital and sport self-efficacy to examine their associations with sport self-handicapping; and Model 4 simultaneously included psychological capital, sport self-efficacy, and sport self-handicapping to examine the associations between the three predictors and sport performance. The complete estimation results of the four regression models are presented in Table 3, and the corresponding models are described below.

**Table 3:** Regression analysis of the chain mediation model.

Predictor variables	Model 1: sport performance (Y)	Model 2: sport self-efficacy (M1)	Model 3: sport self-handicapping (M2)	Model 4: sport performance (Y)
Psychological capital (X)	β = 0.592^***^	β = 0.491^***^	β = −0.376^***^	β = 0.260^***^
	*t* = 14.847	*t* = 11.387	*t* = −8.195	*t* = 6.358
	*f*^2^ = 0.543	*f*^2^ = 0.319	*f*^2^ = 0.166	*f*^2^ = 0.100
Sport self-efficacy (M1)	—	—	β = −0.314^***^	β = 0.294^***^
			*t* = −6.836	*t* = 7.317
			*f*^2^ = 0.115	*f*^2^ = 0.133
Sport self-handicapping (M2)	—	—	—	β = −0.353^***^
				*t* = −8.578
				*f*^2^ = 0.182
Sex	β = 0.001	β = 0.024	β = 0.070	β = 0.016
	*t* = 0.017	*t* = 0.551	*t* = 1.768	*t* = 0.482
	*f*^2^ < 0.001	*f*^2^ = 0.001	*f*^2^ = 0.008	*f*^2^ = 0.001
Age	β = −0.061	β = −0.055	β = −0.022	β = −0.046
	*t* = −1.521	*t* = −1.284	*t* = −0.543	*t* = −1.385
	*f*^2^ = 0.006	*f*^2^ = 0.004	*f*^2^ = 0.001	*f*^2^ = 0.005
*R* ^2^	0.358	0.248	0.358	0.561
Adjusted *R*^2^	0.353	0.243	0.351	0.556
*F*	75.530^***^	44.727^***^	56.398^***^	103.309^***^

β represents the standardized regression coefficient; *f*^2^ represents the local Cohen's *f*^2^ effect size. All models included sex and age as covariates. Multicollinearity diagnostics showed that the VIF values of all predictors ranged from 1.000 to 1.557, and the tolerance values ranged from 0.642 to 1.000, indicating no substantial multicollinearity problems. ^*^p < 0.05, ^**^p < 0.01, ^***^p < 0.001.

Before interpreting each regression path, multicollinearity diagnostics were conducted for all component models. The results showed that the VIF values of predictors across all models ranged from 1.000 to 1.557, and tolerance values ranged from 0.642 to 1.000. Specifically, in Model 4, which simultaneously included psychological capital, sport self-efficacy, sport self-handicapping, sex, and age, the VIF values of all predictors were no greater than 1.557, and tolerance values were no lower than 0.642. Therefore, no substantial multicollinearity problem was identified among predictors, indicating that the subsequent regression coefficients could be interpreted with confidence.

(1) With sport performance as the dependent variable, As shown in Model 1 of Table 3, after controlling for sex and age, psychological capital still significantly positively predicted sport performance (β = 0.592, *t* = 14.847, *p* < 0.001). The model explained 35.8% of the variance in sport performance (*R*^2^ = 0.358, adjusted *R*^2^ = 0.353), and the overall model was significant (*F* = 75.530, *p* < 0.001). The local effect size of psychological capital was *f*^2^ = 0.543, indicating a large effect size. This indicates that psychological capital significantly and positively predicts elite athletes' sport performance; higher levels of psychological capital are associated with better performance in training and competition. Thus, Hypothesis H1 was supported.
(2) With sport self-efficacy as the dependent variable, As shown in Model 2 of Table 3, after controlling for sex and age, psychological capital still significantly positively predicted sport self-efficacy (β = 0.491, *t* = 11.387, *p* < 0.001). The model explained 24.8% of the variance in sport self-efficacy (*R*^2^ = 0.248, adjusted *R*^2^ = 0.243), and the overall model was significant (*F* = 44.727, *p* < 0.001). The local effect size of psychological capital was *f*^2^ = 0.319, indicating a moderate-to-large independent explanatory contribution of psychological capital to sport self-efficacy. This indicates that higher levels of psychological capital are associated with stronger sport self-efficacy in elite athletes. That is, athletes with higher psychological capital are more likely to form positive capability judgments and believe in their ability to complete training tasks, cope with competitive pressure, and control their sport performance. This result provides preliminary evidence for the mediating role of sport self-efficacy in the association between psychological capital and sport performance.
(3) With sport self-handicapping as the dependent variable, As shown in Model 3 of Table 3, after controlling for sex and age, psychological capital still significantly negatively predicted sport self-handicapping (β = −0.376, *t* = −8.195, *p* < 0.001), and sport self-efficacy also significantly negatively predicted sport self-handicapping (β = – 0.314, *t* = −6.836, *p* < 0.001). The model explained 35.8% of the variance in sport self-handicapping (*R*^2^ = 0.358, adjusted *R*^2^ = 0.351), and the overall model was significant (*F* = 56.398, *p* < 0.001). The local effect sizes of psychological capital and sport self-efficacy were *f*^2^ = 0.166 and *f*^2^ = 0.115, respectively. The former reached a medium effect size, whereas the latter showed a small-to-medium effect size, indicating that both variables retained independent explanatory contributions to sport self-handicapping after controlling for demographic covariates and the other predictor. This indicates that elite athletes with higher levels of psychological capital exhibit lower tendencies toward sport self-handicapping. Meanwhile, higher levels of sport self-efficacy are associated with a lower likelihood of athletes adopting self-handicapping strategies such as defensive attribution, reduced effort investment, and avoidance of challenges. Thus, psychological capital is not only directly associated with reduced sport self-handicapping but may also further reduce self-handicapping behaviors by enhancing sport self-efficacy.
(4) With sport performance as the dependent variable, as shown in Model 4 of Table 3, after controlling for sex and age, psychological capital, sport self-efficacy, and sport self-handicapping were simultaneously entered into the regression equation. The results showed that psychological capital significantly positively predicted sport performance (β = 0.260, *t* = 6.358, *p* < 0.001), sport self-efficacy significantly positively predicted sport performance (β = 0.294, *t* = 7.317, *p* < 0.001), and sport self-handicapping significantly negatively predicted sport performance (β = −0.353, *t* = −8.578, *p* < 0.001). The model explained 56.1% of the variance in sport performance (*R*^2^ = 0.561, adjusted *R*^2^ = 0.556), and the overall model was significant (*F* = 103.309, *p* < 0.001). The corresponding local effect sizes of the three predictors were *f*^2^ = 0.100, 0.133, and 0.182, respectively. These results indicated that psychological capital and sport self-efficacy showed small-to-medium independent explanatory effects, whereas sport self-handicapping reached a medium effect size. Therefore, after controlling for sex and age, the three core psychological variables each retained independent and practically meaningful explanatory contributions to sport performance. This indicates that after controlling for sport self-efficacy and sport self-handicapping simultaneously, psychological capital still significantly and positively predicts sport performance, suggesting that sport self-efficacy and sport self-handicapping may play partial (rather than full) mediating roles in the association between psychological capital and sport performance.

### 4.5 Multiple mediation analysis

To further examine the mediating roles of sport self-efficacy and sport self-handicapping in the association between elite sport Psychological Capital and sport performance, this study employed Model 6 in the PROCESS macro to conduct a chain mediation analysis. Elite sport Psychological Capital was used as the independent variable, sport performance as the dependent variable, and sport self-efficacy and sport self-handicapping as the mediating variables, with gender and age included as control variables in the model. A bias-corrected bootstrap method with 5,000 resamples was used, and the significance of indirect effects was determined based on 95% confidence intervals. An indirect effect was considered significant when the confidence interval did not contain zero.

As shown in Table 4, after controlling for sex and age, the total effect of psychological capital on sport performance was 0.4035 (BootSE = 0.0291), with a 95% Bootstrap confidence interval of [0.3467, 0.4589]. Since the confidence interval did not include zero, the total effect was statistically significant. After including sport self-efficacy and sport self-handicapping in the model, the direct effect of psychological capital on sport performance remained significant, with an effect estimate of 0.1777 (BootSE = 0.0318) and a 95% Bootstrap confidence interval of [0.1145, 0.2399]. Meanwhile, the total indirect effect was 0.2259 (BootSE = 0.0224), with a 95% Bootstrap confidence interval of [0.1846, 0.2720], which also did not include zero. This indicates that psychological capital not only indirectly associates with sport performance through the mediating variables but also has an independent direct predictive association with sport performance. Therefore, sport self-efficacy and sport self-handicapping play partial (rather than full) mediating roles in the association between psychological capital and sport performance.

**Table 4:** Total effect, direct effect, and bootstrap indirect effects of the chain mediation model.

Model pathways	Effect estimate	BootSE	BootLLCI	BootULCI
Total effect	0.4035	0.0291	0.3467	0.4589
Direct effect	0.1777	0.0318	0.1145	0.2399
Total indirect effect	0.2259	0.0224	0.1846	0.272
X → M1 → Y	0.0984	0.0157	0.0696	0.1311
X → M2 → Y	0.0904	0.0162	0.0614	0.1245
X → M1 → M2 → Y	0.0371	0.0084	0.0224	0.055

X = psychological capital; M1 = sport self-efficacy; M2 = sport self-handicapping; Y = sport performance. Control variables included sex (female = 0, male = 1) and age. BootSE = Bootstrap standard error; BootLLCI and BootULCI represent the lower and upper limits of the 95% Bootstrap confidence interval, respectively. Bootstrap resampling was conducted with 5,000 iterations; the corresponding effect was considered statistically significant when the confidence interval did not include zero.

Regarding the specific mediation paths, sport self-efficacy significantly mediated the relationship between psychological capital and sport performance. Psychological capital significantly positively predicted sport self-efficacy, and sport self-efficacy further positively predicted sport performance. As shown by the path “X → M1 → Y” in Table 4, the indirect effect was 0.0984 (BootSE = 0.0157), with a 95% Bootstrap confidence interval of [0.0696, 0.1311], which did not include zero, indicating a significant mediation effect. This indirect effect accounted for 24.4% of the total effect. These findings suggest that elite athletes with higher levels of psychological capital generally possess stronger sport self-efficacy, have greater confidence in completing training tasks, coping with competitive pressure, and regulating competitive performance, thereby demonstrating better sport performance. Hypothesis H2 was supported.

As shown by the path “X → M2 → Y” in Table 4, the indirect effect was 0.0904 (BootSE = 0.0162), with a 95% Bootstrap confidence interval of [0.0614, 0.1245], which did not include zero, indicating a significant mediation effect. This indirect effect accounted for 22.4% of the total effect. These results indicate that higher levels of psychological capital are associated with a lower tendency to adopt sport self-handicapping strategies among elite athletes. Lower levels of sport self-handicapping may reduce negative behaviors such as avoidance, excuse-making, and defensive attribution, thereby contributing to improved sport performance. Hypothesis H3 was supported.

As shown by the path “X → M1 → M2 → Y” in Table 4, the chain indirect effect was 0.0371 (BootSE = 0.0084), with a 95% Bootstrap confidence interval of [0.0224, 0.0550], which did not include zero, indicating a significant chain mediation effect. This indirect effect accounted for 9.2% of the total effect. These findings indicate that the influence of psychological capital on sport performance is not achieved only through a single psychological mechanism; rather, it involves a sequential mechanism of “positive capability belief enhancement–defensive strategy reduction–sport performance improvement.” Hypothesis H4 was supported.

## 5 Discussion

### 5.1 The association between psychological capital and sport performance among athletes

H1 proposed that psychological capital positively predicts sport performance among elite athletes. The results showed that psychological capital significantly positively predicted sport performance (β = 0.592, *t* = 14.847, *p* < 0.001), supporting H1. Further analysis showed that, after including sport self-efficacy and sport self-handicapping in the model, the direct effect of psychological capital on sport performance remained significant (Effect = 0.1777, 95% CI [0.1145, 0.2399]), indicating that the relationship between psychological capital and sport performance could not be fully explained by the two mediating variables. In this study, sport performance was measured using an athlete self-report scale; therefore, the “sport performance” discussed here should be understood as athletes' perceived or subjective evaluation of their competitive performance rather than objective competitive indicators such as rankings, competition results, winning or losing outcomes, or other objective performance statistics. The findings support the association between psychological capital and athletes' perceived sport performance, as well as the indirect roles of sport self-efficacy and sport self-handicapping in this association. Because athletes with higher levels of positive psychological capital may also evaluate their competitive status in a more favorable manner, the present findings should not be directly interpreted as evidence that psychological capital inevitably improves objective competition outcomes. This finding supports the fundamental proposition of the psychological capital theory proposed by Luthans et al. (2007), in which psychological capital is conceptualized as a positive psychological state composed of hope, optimism, resilience, and self-efficacy. Such psychological resources are considered beneficial for maintaining positive expectations, sustained effort, and effective adaptation under stressful conditions. For elite athletes, these positive psychological resources represent not only general psychological advantages but also important foundations for maintaining stable sport performance in high-pressure competitive environments. The present findings are also consistent with those reported by Lai et al. (2020). Using a sample of 800 Taiwanese adolescent baseball athletes, significant associations were identified between psychological capital, sport performance, and athletic career development. Compared with their study, the present research focused on elite athletes, further suggesting that the positive association between psychological capital and sport performance may not be limited to adolescent athletes but may also be applicable to athletes at higher competitive levels. In addition, Choi et al. (2025), from the perspectives of emotion regulation and positive psychological capital, reported that positive psychological capital was positively associated with sport performance among athletes, further supporting the present findings.

From the perspective of positive psychological resources, stronger adaptive capacities in response to training and competition-related stress may be facilitated by psychological capital. Athletes with higher levels of hope are more likely to develop clear pathways toward competitive goals and to seek alternative solutions when difficulties are encountered. Athletes with higher levels of optimism are more likely to interpret failure as temporary and adjustable rather than as a complete negation of personal ability. Athletes with stronger resilience are more capable of recovering psychologically after mistakes, injuries, or competition failure and of re-engaging in training. In addition, athletes with higher levels of self-efficacy are more likely to believe in their capability to accomplish competitive tasks and to maintain confidence during critical moments. This interpretation is consistent with the research logic proposed by Yu et al. (2025), in which psychological capital was found to reduce athlete burnout through coping strategies. Psychological capital can thus serve as a source of psychological support for athletes under stress and help them maintain a more positive mind and body during training.

In addition, after adding sport self-efficacy and sport self-handicapping to the model, psychological capital was still a relatively strong positive predictor of sports performance. Based on the above, it can be concluded that while both sport self-efficacy and sport self-handicapping are involved in the pathway linking psychological capital and sports performance, these two factors alone are insufficient to fully account for this relationship. Psychological capital may also be connected to sports performance through the above pathways: emotion regulation, stress coping, attention control, goal persistence and training engagement. In a meta-analysis of the psychological factors and sports performance, Ayranci and Aydin (2025) have found that psychological skills training is needed to improve the performance of athletes, and the links among various psychological factors and sports performance are complex and context-dependent. Therefore, according to the above analysis, psychological capital may be mediated by various psychological pathways.

### 5.2 The mediating role of sport self-efficacy

H2 proposed that sport self-efficacy mediates the relationship between psychological capital and sport performance. After controlling for sex and age, the Bootstrap results showed that the specific indirect effect of the path “psychological capital → sport self-efficacy → sport performance” was 0.0984, with a 95% Bootstrap CI of [0.0696, 0.1311]. Since the confidence interval did not include zero, H2 was supported. This finding supports the core proposition of Bandura's and Wessels (1997) social cognitive theory, according to which individuals' judgments regarding their own capabilities are associated with goal selection, effort expenditure, persistence, and emotional responses. Athletes with higher levels of sport self-efficacy have been shown to respond more rapidly under acute psychological stress, adjust their behaviors more effectively, and attend more strongly to positive information (Guo et al., 2019). This finding is consistent with the mechanism identified in the present study, in which sport performance was promoted through the enhancement of sport self-efficacy by psychological capital. Sport self-efficacy represents not only a subjective belief but also an important factor associated with information processing, behavioral adjustment, and coping quality under stressful conditions.

Psychological Resource Transformation: sport self-efficacy can act as a mediator for the effect of psychological capital on sports performance outcomes. Psychological capital is the positive psychological resources available to athletes, such as hope, optimism and resilience; sport self-efficacy translates these resources into particular belief systems of ability and is expressed as one's belief that one can successfully perform in competitive situations. Athletes with a higher sense of self-efficacy in sports are generally more willing to face difficult training tasks, keep making good decisions and movements during competition, and continue putting in effort even after making mistakes. On the other hand, if there is a lack of sport self-efficacy, positive psychological resources may fail to be converted into stable behavior even when these resources are present. The above results also have some applications. Psychological training for top-tier athletes needs to use positive attitudes and other motivational factors. Improve cultivation of stable, specific and context-bound capability beliefs more rigorously. Through the above methods of phased goal setting, accumulation of successful experiences, simulation of competitive circumstances, positive feedback and technical review, feelings of self-control and accomplishment can be enhanced for the athletes. When the athletes believe that their goals are achievable, they will be motivated to perform better during training, so even if there are mistakes, there is time for correction, and they will face less pressure at the competition level.

### 5.3 The negative mediating role of sport self-handicapping

H3 proposed that sport self-handicapping mediates the relationship between psychological capital and sport performance. After controlling for sex and age, the Bootstrap results showed that the specific indirect effect of the path “psychological capital → sport self-handicapping → sport performance” was 0.0904, with a 95% Bootstrap CI of [0.0614, 0.1245]. Since the confidence interval did not include zero, H3 was supported. This finding supports the classical perspective proposed by Berglas and Jones (1978), according to which individuals may protect self-worth in potentially failure-related performance situations by creating obstacles or providing excuses. For elite athletes, competition outcomes are highly public and evaluative in nature, and failure is often accompanied by evaluations from coaches, teammates, audiences, and the media. Under such high-pressure and highly evaluative conditions, sport self-handicapping may therefore be more easily activated. In the short term, self-handicapping may reduce psychological threats associated with failure; however, in the long term, effort investment may be weakened, authentic feedback may be disrupted, and effective reflection on the causes of failure may be hindered. In addition, Coudevylle et al. (2011) reported that confidence negatively predicted claimed self-handicapping and mediated the association between self-esteem and self-handicapping. The present study further extends this logic to the framework of psychological capital, suggesting that positive psychological resources may promote sport performance through the enhancement of capability beliefs and the reduction of defensive strategies. The existing conclusion that stronger confidence is associated with lower self-handicapping is therefore further supported, while psychological capital may also represent an important upstream psychological resource for reducing sport self-handicapping among elite athletes.

Self-handicapping in sports performance is generally considered to have a negative effect. Athletes with a higher degree of sport self-handicapping are likely to reduce genuine effort during training and competition or psychologically prepare explanatory “exit routes” for potential failure in advance. The behavior may cause loss of focus on learning and application, reducing overall performance. Sports self-handicapping may also prevent accurate determination of the reasons for a poor result and reduce the opportunity to learn from failure. As a result, reduced sport self-handicapping may lead to an improvement in a person's current sporting performance and the promotion of continuous improvement in sports over time. Based on the above results, psychological support for top-tier athletes should also target the cultivation of positive psychology by modifying defensive psychological habits. Coaches and sport psychology professionals should observe for behaviors such as excuse-making, challenge avoidance, decreased effort investment and externalization of blame during training and competition. Attribution training, failure review, cognitive restructuring and psychological support can all be used to reduce sport self-handicapping.

### 5.4 The chain mediating role of sport self-efficacy and sport self-handicapping

H4 proposed that sport self-efficacy and sport self-handicapping form a chain mediation effect between psychological capital and sport performance. After controlling for sex and age, the Bootstrap results showed that the chain indirect effect of the path “psychological capital → sport self-efficacy → sport self-handicapping → sport performance” was 0.0371, with a 95% Bootstrap CI of [0.0224, 0.0550]. Since the confidence interval did not include zero, H4 was supported. These findings reveal a continuous psychological mechanism underlying the association between psychological capital and sport performance, namely: “positive psychological resources → enhanced capability beliefs → reduced defensive strategies → improved sport performance.”

In general, psychologically motivated people are more willing to believe that they can achieve high levels of self-actualisation in their sports. Athletes with a high level of psychological capital generally have strong hope, optimism and resilience; therefore, a more positive sense of self-efficacy and a stronger perception of controllability are more likely to be maintained in the face of training difficulties and competition pressure. The above positive psychological resources can increase athletes' confidence in themselves and improve their belief in performing well in sports by helping them cope with training difficulties and competition pressures; thus, they are able to boost self-efficacy. Then, higher sports self-efficacy can further reduce sport self-handicapping. When athletes believe that they are capable of handling problems, they are not in a state of low self-esteem and will feel less pressure to reduce effort, make excuses or delay preparation due to the fear of failure. Finally, a reduction in sport self-handicapping may help concentrate more on the technical execution, tactical adjustment and goals of play to improve one's own sports ability. The above results are also feasible. The chain mechanism identified in this study should first be understood as a finding within the context of Chinese professional competitive sports. Factors such as collective goals, coach authority, and social evaluation in Chinese competitive sports may influence athletes' ability judgments and coping styles in response to failure (Si et al., 2015). However, because no cross-cultural comparison was conducted, it cannot be assumed that this pathway operates similarly across different cultural groups. The main findings remained stable after controlling for age and sex, but sport type was not included in the primary model; therefore, the potential influence of sport-specific differences cannot be completely ruled out. Psychological Training for elite athletes should cover the three levels simultaneously. First, foster psychological capital to strengthen positive psychological resources such as hope, optimism, resilience and self-efficacy. Second, boost students' sports self-efficacy through successful experiences, goal decomposition, positive feedback and situation simulation to build stronger self-confidence in their ability. Third, reduce sport self-handicapping by using attribution training, failure review and cognitive restructuring, and thus reduce excuse-making and avoidant behavior in athletes. Only when the positive psychological resources have been transformed into ability beliefs and defense-oriented psychological behavior can they be further inhibited; then, the promoting effect of psychological capital on sports performance can be fully achieved.

## 6 Limitations and future directions

This study examined the relationship between elite sport Psychological Capital and their performance in sports, as well as the chain-mediating effect of sport self-efficacy and sport self-handicapping, and obtained some positive results. There are also some defects. First, this study used a cross-sectional design and did not employ a longitudinal approach; thus, a causal relationship could not be identified. Second, this study adopted a non-probability sampling method, and participants were mainly recruited from provincial-level and above professional sports teams across five provinces. Although multi-regional sampling increased the heterogeneity of participant sources to some extent, the absence of nationwide probability random sampling means that some selection bias may still exist. Therefore, the external generalizability of the findings should be interpreted with caution. Future studies may adopt stratified random sampling, multi-stage sampling, or larger-scale multicenter research designs across broader regions and different competitive sports to further examine the stability and generalizability of the present findings. Third, the core variables in this study were mainly obtained through self-reports collected at the same time point and may therefore still be influenced by common method bias and social desirability. Psychological capital, sport self-efficacy, and self-reported sport performance all involve relatively positive self-evaluative attributes; participants' tendency to provide favorable responses may increase shared variance and inflate correlations among positive constructs. Sport self-handicapping represents a relatively negative self-evaluative behavior and may be underreported, thereby reducing variance and potentially biasing correlation estimates. If multiple negative constructs are included in future studies, common underreporting may also weaken correlations among negative constructs; however, this pattern could not be directly examined in the present study. Future research may include social desirability scales and incorporate coach evaluations, behavioral observations, and multi-source data. Fourth, sport performance in this study was measured using an athlete self-report scale; therefore, sport performance mainly reflects individuals' subjective perceptions of their competitive state and performance quality rather than objective competition outcomes. Although subjective performance evaluations can capture athletes' overall experiences of plan execution, emotional control, fluency, and tactical execution, they may still be influenced by individual self-evaluation tendencies and psychological states. Therefore, the associations identified in this study among psychological capital, sport self-efficacy, sport self-handicapping, and perceived sport performance should not be directly generalized to objective competitive performance. Future studies may combine official competition results, competition rankings, winning and losing outcomes, sport-specific technical statistics, coach evaluations, or other objective performance indicators with multi-source methods to examine whether the chain mediation mechanism proposed in this study can also explain objective competitive performance. Fifth, although age and sex were controlled in the primary analyses, sport type was not further included in the regression and chain mediation models. Future studies may adopt more detailed classifications of sport disciplines after obtaining larger samples with sufficient representation across different sports, and examine the stability of the proposed chain mechanism across different sport types and specific disciplines through covariate control, stratified analyses, or multi-group structural equation modeling.

On this basis, future research can extend the chain framework proposed in this study in three directions. First, individual psychological mechanisms can be integrated with team knowledge processes, team faultlines, and collaborative norms, and multilevel models can be adopted to examine individual–team cross-level interactions (Shahzad et al., 2023; Jiang et al., 2012, 2022). Second, future research can examine how organizational environments shape the process of “psychological capital–sport self-efficacy–sport self-handicapping–sport performance” by considering leadership behaviors, coach feedback, trust, training management practices, and stakeholder factors as antecedents or boundary conditions (Shao et al., 2023; Guo et al., 2024; Tang et al., 2018; Wang et al., 2026). Third, future studies can examine moderating factors such as environmental perceptions, leadership autonomy support, competitive culture, and intercultural competence, and extend this framework to physical education, exchange athletes, or transnational training contexts to further test its applicability boundaries across cultures and performance domains (Cheng et al., 2022; Wang et al., 2024; Peng et al., 2026).

## 7 Conclusions

(1) Elite sport Psychological Capital has a significant positive association with sport performance. This suggests that psychological capital is not merely a general positive psychological state but an important psychological resource that directly associates with competitive performance. (2) Sport self-efficacy plays a mediating role in the association between psychological capital and sport performance. Psychological capital significantly enhances elite athletes' sport self-efficacy, which further facilitates sport performance. (3) Sport self-handicapping plays a negative mediating role in the association between psychological capital and sport performance. Psychological capital significantly reduces elite athletes' tendency toward sport self-handicapping, and lower sport self-handicapping further facilitates sport performance. (4) Sport self-efficacy and sport self-handicapping formed a significant chain mediation path, indicating that psychological capital was first associated with higher sport self-efficacy, subsequently associated with lower sport self-handicapping, and ultimately associated with better sport performance. The findings of this study support a pattern in which psychological capital is directly associated with sport performance while also showing multiple indirect associations through specific psychological mechanisms.

## Data Availability

The raw data supporting the conclusions of this article will be made available by the authors, without undue reservation.
